# Chaperones Skp and SurA dynamically expand unfolded OmpX and synergistically disassemble oligomeric aggregates

**DOI:** 10.1073/pnas.2118919119

**Published:** 2022-02-25

**Authors:** Neharika Chamachi, Andreas Hartmann, Mai Quynh Ma, Anna Svirina, Georg Krainer, Michael Schlierf

**Affiliations:** ^a^B CUBE - Center for Molecular Bioengineering, Technische Universität Dresden, 01307 Dresden, Germany;; ^b^Yusuf Hamied Department of Chemistry, Centre for Misfolding Diseases, University of Cambridge, CB2 1EW Cambridge, United Kingdom;; ^c^Cluster of Excellence Physics of Life, Technische Universität Dresden, 01062 Dresden, Germany

**Keywords:** chaperones, outer membrane protein biogenesis, single-molecule FRET, protein folding, disaggregation

## Abstract

Periplasmic chaperones 17-kilodalton protein (Skp) and survival factor A (SurA) are essential players in outer membrane protein (OMP) biogenesis. They prevent unfolded OMPs from misfolding during their passage through the periplasmic space and aid in the disassembly of OMP aggregates under cellular stress conditions. However, functionally important links between interaction mechanisms, structural dynamics, and energetics that underpin both Skp and SurA associations with OMPs have remained largely unresolved. Here, using single-molecule fluorescence spectroscopy, we dissect the conformational dynamics and thermodynamics of Skp and SurA binding to unfolded OmpX and explore their disaggregase activities. We show that both chaperones expand unfolded OmpX distinctly and induce microsecond chain reconfigurations in the client OMP structure. We further reveal that Skp and SurA bind their substrate in a fine-tuned thermodynamic process via enthalpy–entropy compensation. Finally, we observed synergistic activity of both chaperones in the disaggregation of oligomeric OmpX aggregates. Our findings provide an intimate view into the multifaceted functionalities of Skp and SurA and the fine-tuned balance between conformational flexibility and underlying energetics in aiding chaperone action during OMP biogenesis.

## Significance

Outer membrane proteins (OMPs) are crucial for the survival of bacteria. The two chaperones 17-kilodalton protein (Skp) and survival factor A (SurA) play key roles in OMP maturation by keeping unfolded OMP proteins soluble in the periplasm. However, their functionalities are incompletely understood. Here, we establish connections between structural and energetic features employed by the two chaperones when interacting with unfolded OmpX. We find that expansion, accompanied with fast polypeptide chain reconfiguration, prevents unfolded OmpX from misfolding and aggregating. Moreover, chaperone interaction with unfolded OmpX is thermodynamically calibrated, allowing for a fine-tuned association of chaperones with OMPs in the adenosine triphosphate-depleted periplasm. We further discovered that Skp and SurA act together as disaggregases and are able to disassemble oligomeric OMP aggregates, revealing remarkable functionalities of this periplasmic chaperone system.

Molecular chaperones are key cellular components that play fundamental roles in maintaining cellular proteostasis (1, 2). Essential activities of chaperones include the assistance of de novo protein folding, the stabilization of nonnative proteins in folding competent or unfolded states, and the rescue of misfolded and aggregated proteins (3–5). Chaperones are an integral part of a wide range of protein quality-control systems, and their activities are intimately coupled to the biogenesis networks that aid the structural and functional maturation of proteins from their site of cellular synthesis to their target cellular compartments.

One network where chaperone activity is of particular relevance is the biogenesis of outer membrane proteins (OMPs) (6, 7). OMPs are a diverse group of β-barrel membrane proteins found in the outer membrane of Gram-negative bacteria, mitochondria, and chloroplasts. They fulfill a plethora of functions in cell signaling, metabolism, and transport (8–10); are indispensable to the survival of bacteria (10, 11); and constitute important virulence factors and drug targets (12–14). The OMP biosynthesis pathway is highly complex and conserved across all kingdoms of life (15) and involves the coordinated action of a multicomponent protein machinery that aids in overcoming the many hurdles that these proteins have to surmount on their way to their target outer membrane (4, 7, 16).

In Gram-negative bacteria, OMPs are translated in the cytoplasm, from where they are translocated across the inner bacterial membrane via the Sec machinery to the periplasmic space (17, 5). Within this aqueous compartment, OMPs are escorted to the outer membrane in an unfolded state (denoted as the uOMP state) with the aid of various chaperones that maintain the largely insoluble and aggregation-prone uOMP polypeptide chains in a protected, partially unfolded state (18, 19). At the outer membrane, uOMPs are transferred to the β-barrel assembly machinery (BAM), which facilitates their native folding and insertion into the membrane (20). Noteworthy, the periplasm is devoid of any known source of energy-providing molecules, such as adenosine triphosphate (ATP); hence, all chaperones as well as the entire folding machinery likely operate without the aid of external energy, following thermodynamic principles (21).

Two chaperones which have been shown to be indispensable for the biogenesis of bacterial OMPs are the 17-kilodalton protein (Skp) (22) and survival factor A (SurA) (23). Skp and SurA, both located in the periplasm, exhibit antifolding activity (also known as holdase activity), whereby they sequester uOMP substrates to prevent aggregation until they reach the bacterial outer membrane (24–26). Their interaction with uOMPs is thermodynamically modulated due to the lack of energy-carrying molecules in the periplasm (27–29). Depletion studies of periplasmic chaperones identified SurA as an essential chaperone for OMP biogenesis, leading to a drastic decrease in OMP density in the outer membrane due to the loss of SurA (30). Skp depletion, on the other hand, led to an accumulation of misfolded OMPs and the activation of the cellular stress response (30), while OMP density in the outer membrane remained the same. Interestingly, recent studies suggest substrate selectivity among the two chaperones (31). Hence, it is of importance to understand the functional mechanisms underlying both Skp and SurA association with uOMPs.

Structural studies of the eight β-stranded protein outer membrane protein X (OmpX) in the presence of Skp using NMR spectroscopy have found that unfolded OmpX (uOmpX) shows submillisecond backbone dynamics (32) in complex with Skp. For binding of SurA to unfolded outer membrane protein A (uOmpA), both fluid globular (32) and expanded states (31) have been proposed. Recent studies have located various interaction sites of SurA and uOmpX using cross-linking, suggesting that SurA-bound uOmpX populates multiple conformations (31, 33). Yet, long-range polypeptide chain dynamics and conformational heterogeneity of unfolded OMPs upon binding to chaperones remain elusive. In particular, it is unknown how SurA- and Skp-bound OMP dynamics and heterogeneities differ, given their differential roles in regulating protein folding in the periplasmic space. Dynamic aspects are hypothesized to be important for chaperone–uOMP interactions, particularly to fine-tune energetics of the binding reaction through a reduction of the entropic costs upon binding (28, 29, 34–37). Yet, the enthalpic and entropic changes that determine Skp–OMP or SurA–OMP interactions and affect the conformations of the denatured substrate proteins are largely undefined.

In addition to the well-described holdase activities of Skp and SurA that protect OMPs from misfolding or aggregating, a recent study has suggested that Skp disaggregates oligomeric uOMP structures (38). While SurA has not been directly implicated as a disaggregase, modeling studies propose a synergistic interaction among these chaperones especially under conditions of stress (18, 39), thus raising the question of the role that both chaperones played in disassembling OMP aggregates.

To gain insights into the multifaceted functionalities of Skp and SurA and their action mechanisms, we study here the conformational dynamics and thermodynamics of the eight β-stranded protein OmpX in the presence of the chaperones and explore their disaggregation activities. Using single-molecule Förster resonance energy transfer (smFRET), we resolve the heterogeneities, structural dynamics, and thermodynamics underlying the different states of uOmpX at near-native conditions. Strikingly, we find that both chaperones expand the unfolded polypeptide chain upon binding. The degree of expansion is concentration dependent for SurA, but not for Skp. Probing structural changes and chaperone interaction at different temperatures, we gain insights into the enthalpic and entropic contributions of complex formation and find that the interaction modes of both chaperones differ strongly and are dictated by entropy–enthalpy compensation. Finally, we use fluorescence correlation spectroscopy (FCS) to probe the disaggregation capabilities of Skp and SurA and find synergistic activity of both chaperones in the disassembly reaction of oligomeric OmpX aggregates. Our findings provide fundamental insights into the structural and energetic mechanisms underlying Skp and SurA chaperone–OMP interactions and their role in OMP biogenesis.

## Results

### Studying Chaperone Effects on uOmpX Conformational Dynamics under Native-Like Conditions by smFRET.

Probing chaperone effects and conformational dynamics of uOMPs under native-like conditions (i.e., in the absence of or at very low denaturant concentrations) has been difficult to achieve due to the aggregation-prone nature of OMPs in aqueous environments (4, 7, 40). Single-molecule methods, in particular single-molecule fluorescence techniques such as smFRET, operate at very low concentrations (i.e., in the pM range) and thus alleviate protein aggregation and precipitation challenges (41–43). smFRET measurements further provide access to subpopulation-specific conformational heterogeneity, even at very low denaturant concentrations or the absence thereof (44) and allow probing of long-range polypeptide chain dynamics across a wide spectrum of timescales in the presence of binding partners and without the need of synchronizing the ensemble with perturbation/relaxation techniques (45–47). We therefore set out to develop an smFRET assay that allowed us to study the conformational dynamics of single uOmpX polypeptide chains in native-like aqueous environments and the effect that the chaperones Skp and SurA have on the structural and dynamic properties of uOmpX.

**Fig. 1.**
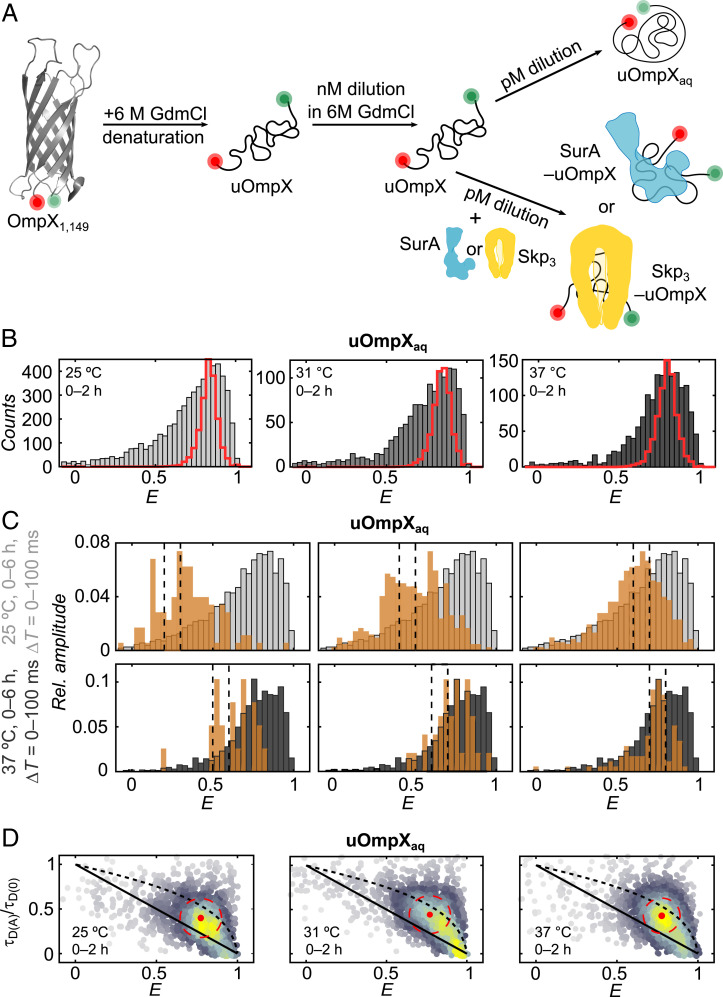
Probing structural dynamics of uOmpX under native-like conditions by smFRET. (*A*) Dilution scheme used for smFRET experiments to study unfolded and chaperone-bound uOmpX. The positions of donor (green sphere) and acceptor (red sphere) fluorophores at the N- and C-terminal ends of OmpX_1,149_ are indicated. (*B*) FRET efficiency (*E*) histograms of uOmpX in aqueous buffer conditions without denaturant (uOmpX_aq_) obtained during the first 2 h of measurement at 25 °C, 31 °C, and 37 °C. Shot noise limited distributions are shown as red cityscapes. (*C*) Recurrence analysis with recurrence time intervals Δ*T* = {0, 100} ms and varying initial Δ*E* windows of 0.2 to 0.3, 0.4 to 0.5, and 0.6 to 0.7 at 25 °C (*Top*, black dashed lines) and 0.5 to 0.6, 0.6 to 0.7, and 0.7 to 0.8 at 37 °C (*Bottom*, black dashed lines). The probability of observing recurrence of the same molecule in the interval of 0 to 100 ms is 81% and 78% for uOmpX_aq_ measurements performed at 25 °C and 37 °C, respectively. Recurrence histograms are shown in orange. Complete FRET efficiency histograms created from all detected bursts are shown in gray. (*D*) 2D scatter plot of relative fluorescence lifetime (τ_D(A)_/τ_D(0)_) vs. *E* for uOmpX_aq_ at 25 °C, 31 °C, and 37 °C for 0 to 2 h of measurement. Lines represent the static FRET line (solid black line) and the expected correlation for a Gaussian chain (dashed black line). The red dot and the red dashed circle denote the center position and 68% area of the uOmpX_aq_ population. The scatter plot density is color coded (gray to yellow).

To begin, we prepared an N- and C-terminal fluorescently labeled double-cysteine OmpX variant (OmpX_1,149_) furnished with donor and acceptor dyes (Fig. 1*A* and *Methods*). The placement of the FRET-dye pair at the terminal ends of the protein allowed us to monitor the conformational properties of the uOmpX polypeptide chain. After preparation, labeling, and refolding of the protein in presence of the surfactant lauryldimethylamine-*N*-oxide (LDAO) (*SI Appendix*, Fig. S1 *A* and *C* and *Methods*), we subjected OmpX_1,149_ to a multistep denaturation and dilution protocol to transfer it to a micelle-free aqueous buffer environment at picomolar concentrations (Fig. 1*A*). Briefly, we first denatured refolded OmpX_1,149_ with 6 M guanidinium chloride (GdmCl) at micromolar concentrations and subsequently diluted it in the presence of 6 M GdmCl to a concentration of 20 nM. This was followed by a 1,000-fold dilution step into GdmCl-free buffer or into GdmCl-free buffer supplemented with the chaperones Skp or SurA. The resultant [GdmCl] after the final dilution step was 6 mM and the remaining [LDAO] was <100 nM, thus yielding uOmpX at 20 pM in a micelle-free native-like aqueous buffer that is largely devoid of denaturant (denoted as uOmpX_aq_). smFRET measurements were performed directly after the dilution protocol, thus mimicking the scenario when newly secreted OMPs enter the periplasm and encounter their interacting chaperones. Subsequently, fluorescence bursts were recorded from a large number of individual protein molecules diffusing through the confocal detection volume to generate FRET efficiency (*E*) histograms (*Methods*). Because smFRET provides intramolecular distance information in the nanometer range by measuring the energy transfer between fluorescent donor and acceptor dyes attached to the polypeptide chain, *E* histograms report on the diversity of conformations with different levels of compactness (i.e., distance between the two dyes) and provide information on intrachain dynamics (43, 48).

### uOmpX in the Absence of Chaperones Is Structurally Heterogeneous and Exhibits Multitier Dynamics.

Under native-like buffer conditions and in the absence of chaperones, the *E* histogram of uOmpX_aq_ at 25 °C in the first 2 h of measurement exhibited a broad distribution with a FRET efficiency peak of the aqueous uOmpX state (*Ê*_aq_) located at *Ê*_aq_ ∼ 0.77 and a tail spreading toward low transfer efficiencies (Fig. 1*B*, *Left*). Broadened *E* distributions are generally ascribed to conformational heterogeneity with interconversion dynamics on timescales comparable to or longer than the observation time (∼1 to 2 ms). Indeed, the width of the unfolded state population is in excess of that expected for a distribution that is limited only by detection shot noise (red cityscape in Fig. 1*B*), indicating the existence of a heterogeneous ensemble of conformations. Even at elevated temperatures of 31 °C and 37 °C (Fig. 1*B*, *Center* and *Right*, respectively), we observed a broadened *E* distribution further indicative of a rough energy landscape of the uOmpX_aq_ state (34). Additionally, recurrence analysis (49) revealed slow interconversions among the various substates on timescales longer than the burst duration (≥100 ms; Fig. 1*C*). This suggests collective and transient conformational changes of remaining secondary motifs or tertiary long-range interactions, as have been described before for other OMPs under denaturing conditions (34, 50). Fluorescence lifetime and sufficient rotational averaging allowed us to exclude the possibility that the broadening originates from changes in dye quantum yields or a restricted rotational freedom of the dyes (*SI Appendix*, Table S1). We also observed a modest expansion of the uOmpX_aq_ chain on the timescale of 4 to 6 h (*SI Appendix*, Results, Fig. S1 *D*–*H*, Table S2), in line with reports which suggest that this mechanism prevents aggregation in the absence of both a membrane mimetic environment and chaperones (40). Thus, our results indicate that uOmpX_aq_ might also possess an intrinsic nonaggregating ability.

In addition to the slow interconversion dynamics that give rise to broadened uOmpX_aq_ distributions, the unfolded polypeptide chain exhibited fast submillisecond reconfiguration dynamics at early time points (0 to 2 h), as indicated by a displacement of the uOmpX_aq_ population from the static FRET line in the relative donor lifetime (τ_D(A)_/τ_D(0)_, i.e., the ratio of the fluorescence lifetime of the donor in the presence and absence of acceptor, respectively) versus *E* plot (Fig. 1*D*). The uOmpX_aq_ population, however, did not fall onto a line that describes the intrachain distance distribution of a Gaussian chain, used to model completely unfolded polypeptides (51) (Fig. 1*D*, dashed line). This substantiates our recurrence analysis (49), in that, uOmpX_aq_ does not behave like a fully unfolded, random coil polypeptide. uOmpX_aq_ thus exhibits a multitier dynamical character with fast and slow intrachain motions, as has been previously observed for other proteins and membrane proteins (52–54).

Interestingly, for the measurement conducted at 25 °C, at late time points (4 to 6 h), the *E* population of uOmpX_aq_, coincided with the static FRET line (*SI Appendix*, Fig. S1*I*, *Left*), indicating that uOmpX_aq_’s fast reconfiguration dynamics vanish over time. This points toward the formation of long-range interactions in the unfolded-state and a dedynamization of the uOmpX_aq_ polypeptide chain while preserving the broad conformational heterogeneity. Such a behavior was absent in the case of the measurement performed at 37 °C (*SI Appendix*, Fig. S1*I*, *Right*) and was reversible upon temperature increase from 25 °C to 37 °C (*SI Appendix*, Fig. S1*J*), likely due to temperature-induced chain dynamics. Of note, in all experiments, an additional minor population (∼10%) at a high FRET efficiency (*E* ∼ 0.92) was observed. This very compact state is particularly observed after long measurement times of 4 to 6 h (*SI Appendix*, Fig. S1*I*) and likely constitutes a misfolded state, denoted as uOmpX_compact_ throughout the article (*SI Appendix*, Fig. S2*A*).

In conclusion, we find that uOmpX_aq_ adopts a broad, heterogeneous ensemble of structures and populates a rugged energy landscape leading to a multitier dynamical behavior with both fast submillisecond chain reconfiguration dynamics and slow conformational interconversion. An overall expansion of the uOmpX_aq_ polypeptide chain is observed, in line with a conformational loosening mechanism that likely serves to alleviate uOMP aggregation (40).

### Skp- and SurA-Bound uOmpX Is Dynamically Expanded.

Next, we probed the effects that the chaperones Skp and SurA have on the structural and dynamic properties of the uOmpX polypeptide chain. To this end, we subjected denatured OmpX to a multistep dilution and denaturation protocol to transfer the protein into native-like buffer in the presence of the chaperones Skp or SurA and subsequently recorded *E* histograms (Fig. 1*A*).

**Fig. 2.**
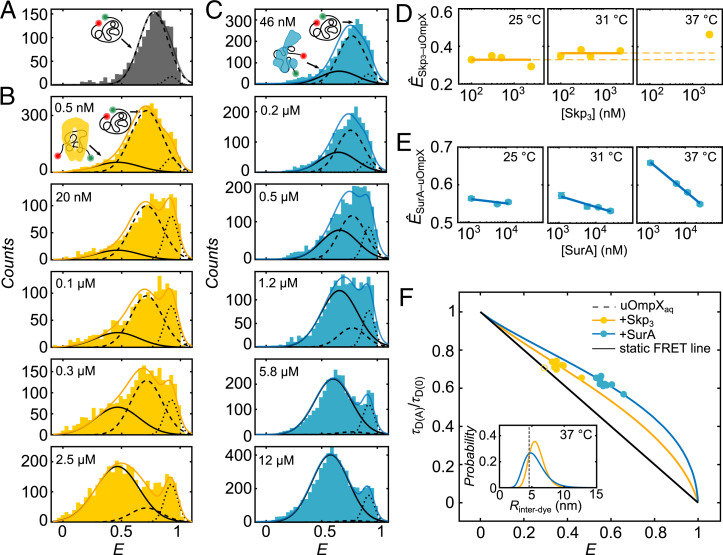
Conformations and structural dynamics of Skp- and SurA-bound uOmpX. (*A*) FRET efficiency (*E*) histogram of uOmpX in the absence of chaperones. (*B* and *C*) *E* histograms of uOmpX in the presence of different concentrations of Skp_3_ (yellow) and SurA (blue), respectively. Concentrations are indicated. The three underlying Gaussian distributions are highlighted by black lines as follows: Skp_3_- or SurA-bound uOmpX (Skp_3_–uOmpX or SurA–uOmpX, solid line), unbound uOmpX (uOmpX_aq_, dashed line), and compact uOmpX (uOmpX_compact_, dotted line). The sum of the three Gaussian distributions is shown in red. FRET state peak positions (*Ê*) of (*D*) Skp_3_–uOmpX and (*E*) SurA–uOmpX at three different temperatures for measurements performed with [Skp_3_] > 100 nM and [SurA] > 1,160 nM, respectively. (*F*) Relative fluorescence lifetime (τ_D(A)_/τ_D(0)_) and *E* position of Skp_3_–uOmpX and SurA–uOmpX (yellow and blue, respectively) populations for different temperatures. Here, we modeled the interdye distance with a log-normal distribution. The black line depicts the static FRET line. The inset shows the average interdye distance ⟨*R*_interdye_⟩ of uOmpX_aq_ (gray dashed line) and the empirical log-normal interdye distance distributions of uOmpX complexed with 2.5 µM Skp_3_ (yellow curve) and 11.6 µM SurA (blue curve), respectively.

In a first set of experiments, we complexed uOmpX with Skp at 37 °C, which binds unfolded OMPs as a trimer (55, 56), denoted hereafter as Skp_3_. As a reference, we first performed a measurement in the absence of the chaperone and again observed a broad distribution at intermediate *E* values, representing the heterogeneous unfolded conformational ensemble of uOmpX_aq_, and a minor compact population at *E* values, representing uOmpX_compact_ (Fig. 2*A*; c.f. Fig. 1*B*). Upon the addition of Skp_3_ to uOmpX, strikingly, an additional population at lower FRET efficiencies (*Ê* ∼ 0.4) gradually arose, being already visible at 0.5 nM [Skp_3_] (Fig. 2*B*). At [Skp_3_] of ∼2.5 µM, which is close to the reported periplasmic concentration of Skp (18, 57, 58), the FRET efficiency distribution was dominated by this low *E* peak. The emergence of a third population at low FRET efficiencies, in addition to the two states of uOmpX in the absence of Skp_3_ (c.f. Fig. 2*A*), suggests that Skp_3_ interacts with uOmpX and forms a chaperone-bound state, in the following denoted as Skp_3_–uOmpX. This state is in equilibrium with and appears structurally distinct and more expanded than the uOmpX_aq_ and uOmpX_compact_ states.

To extract populations from the FRET efficiency histograms, we found that three Gaussian distributions were sufficient to describe the overall shape and change of shape upon chaperone titration (see *SI Appendix*, Fig. S3 and *Methods*). Gaussian fitting showed that the peak positions of the two populations in the high FRET regime exhibited *E* values of *Ê* ∼ 0.75 and *Ê* ∼ 0.92, which are in very good agreement with the *E* values of the uOmpX_aq_ (*Ê*_aq_ ∼ 0.77) and uOmpX_compact_ (*Ê_compact_* ∼ 0.92) states. This suggests that the presence of Skp_3_ does not affect the conformational properties of these states and implies that these do not directly interact with Skp_3_. We therefore identify these states, even in presence of chaperones, as uOmpX_aq_ and uOmpX_compact_ (*SI Appendix*, Fig. S2*B*). The FRET efficiency peak of Skp_3_-bound uOmpX was centered around *Ê*_Skp3–uOmpX_ ∼ 0.45, a considerably larger interfluorophore distance than the unbound uOmpX (i.e., uOmpX_aq_) state. A careful analysis of the donor lifetime, the fluorophore anisotropies, and the rotational correlation times of the fluorophores reassured that the lower *E* values reflect a global conformational change arising from the increased end-to-end distance of the uOmpX polypeptide chain upon binding to Skp_3_ (*SI Appendix*, Table S1). Moreover, Gaussian fitting revealed that, at [Skp_3_] = 2.5 µM, ∼80% of uOmpX molecules were in a Skp_3_–uOmpX complexed state, suggesting that the presence of Skp shifts the equilibrium from the unbound to the bound form.

In a second set of experiments, we diluted uOmpX in the presence of the chaperone SurA at 37 °C. Upon addition of SurA, we observed, similarly to Skp_3_, an additional FRET efficiency peak at lower *E* values (Fig. 2*C*). Increasing [SurA] from 46 nM to 25 µM lead to an increase of the low *E* state population (i.e., SurA–uOmpX). Gaussian fitting allowed us to identify the peak position and fraction of SurA-bound uOmpX with *Ê*_SurA–uOmpX_ ∼ 0.6 at a concentration of ∼6 µM, which is close to the intracellular concentration of SurA (18, 58). At this concentration, ∼87% of uOmpX molecules are bound to the chaperone. The lower *Ê* value of SurA–uOmpX also indicated an expanded conformation as compared to uOmpX_aq_ and yet a less expanded conformation as compared to Skp_3_–SurA. The distinct peak positions of the two chaperone-bound states (*Ê*_Skp3–uOmpX_ ∼ 0.45 and *Ê*_SurA–uOmpX_ ∼ 0.6) thus suggest that they themselves occupy a distinguishable configurational space likely due to the difference in the interaction mechanisms between the chaperone and its substrate (7, 16).

Since temperature affected the conformation and reconfiguration dynamics of uOmpX_aq_, we asked how temperature influences the structural ensemble and dynamics of chaperone-complexed uOmpX. To this end, we performed titration experiments at 31 °C and 25 °C and evaluated *Ê*_Skp3–uOmpX_ and *Ê*_SurA–uOmpX_ at different [Skp_3_] and [SurA] (*SI Appendix*, Fig. S4 and Table S3). The FRET efficiencies of the unbound (uOmpX_aq_) and compact uOmpX (uOmpX_compact_) states did not vary markedly across the chaperone concentration series performed at different temperatures (*SI Appendix*, Figs. S5 and S2 *A*–*C*). For the chaperone-bound fraction of uOmpX, *Ê*_Skp3–uOmpX_ remained constant, within error, across nearly three orders of magnitude of [Skp_3_] at 25 °C (Fig. 2*D*, *Left*). This suggests a concentration-independent, possibly 1:1 stoichiometry, of Skp_3_ and uOmpX, in accordance with previous studies (32, 59). Increasing the temperature of the complex, however, shifted *Ê*_Skp3–uOmpX_ to higher *E* values (Fig. 2*D*), while at each temperature, *Ê*_Skp3–uOmpX_ itself remained constant again across all concentrations. We infer that, at elevated temperatures, uOmpX in complex with the chaperones appears to form a more compact structural ensemble, possibly due to the increased dynamics of uOmpX in the Skp_3_ cavity at 37 °C as compared to 25 °C, similar to recent observations made by NMR spectroscopy (32). By contrast, for SurA–uOmpX complexes, *Ê*_SurA–uOmpX_ decreased with increasing chaperone concentrations at 25 °C (Fig. 2*E*, *SI Appendix*, Table S3). Interestingly, at elevated temperatures, the dependency on [SurA] was enhanced and we observed a strong decrease of *Ê*_SurA–uOmpX_ with increasing [SurA] at 37 °C from *Ê*_SurA–uOmpX_ (1.2 µM) ∼ 0.66 to *Ê*_SurA–uOmpX_ (25 µM) ∼ 0.55. This suggests that uOmpX is more expanded at elevated [SurA], which can be explained by either a different interaction mode or that uOmpX is sequestered by multiple SurAs as also suggested by previous studies (31, 33).

Next, we modeled the structural ensemble of the states globally using a log-normal distribution and obtained a standard deviation (SD) of the donor–acceptor distance (*σ_R_*) and the expected interdye distance ⟨*R*_interdye_⟩ (Fig. 2*F*, inset; *Methods*). A measure of heterogeneity among a population of varying end-to-end distance is the coefficient of variance, $\text{CV}=\sqrt{e^{\sigma_{R}^{2}}–1\;}$, where a larger CV indicates an increased heterogeneity. We determined CVs of 0.204 ± 0.036 and 0.304 ± 0.022 for Skp_3_-bound and SurA-bound uOmpX, respectively. Compared to SurA–uOmpX, the CV of the Skp_3_–uOmpX complex is reduced and indicates less conformational heterogeneity for the bound substrate, likely due to the binding of uOmpX in the cavity of Skp_3_ (55, 56). By contrast, the higher CV for the SurA–uOmpX complex marks an increased heterogeneity. This increase in heterogeneity could originate from the transient multisite binding of SurA resulting in diverse unstructured and expanded uOmpX conformations with none, one, or even multiple SurAs bound. We further used the log-normal distribution to extract interdye distances in order to quantify the end-to-end distances of the chaperone-bound uOMP complexes. Compared to uOmpX_aq_, the interdye distance grows from ${\left\langle R_{\text{interdye}}\right\rangle }_{{\text{uOmpX}}_{\text{aq}}}$ ∼ 4.6 nm to ${\left\langle R_{\text{interdye}}\right\rangle }_{{\text{Skp}}_{3}\text{-uOmpX}}$ ∼ 5.8 nm at 2.5 μM [Skp_3_] and to ${\left\langle R_{\text{interdye}}\right\rangle }_{\text{SurA-uOmpX}}$ ∼ 5.4 nm at 12 μM [SurA].

In a last step, to gain further insights into the structural and dynamical properties of chaperone-bound uOmpX, we analyzed the underlying dynamics of the chaperone-complexed uOmpX polypeptide chain. To this end, we extracted single-molecule events of the chaperone-bound population using a combined FRET efficiency and relative donor fluorescence lifetime filter (*SI Appendix*, Fig. S3). We determined the peak positions for *E* and τ_D(A)_/τ_D(0)_ for each condition and plotted the values in a two-dimensional (2D) diagram (Fig. 2*F* and *SI Appendix*, Tables S3 and S4). The bound FRET efficiencies (*Ê*_Skp3–uOmpX_ and *Ê*_SurA–uOmpX_) across different chaperone concentrations were offset in distinct clusters from the static FRET line. The offset indicates that the polypeptide chain undergoes fast configurational changes on the submillisecond timescale while being bound to chaperones.

**Fig. 3.**
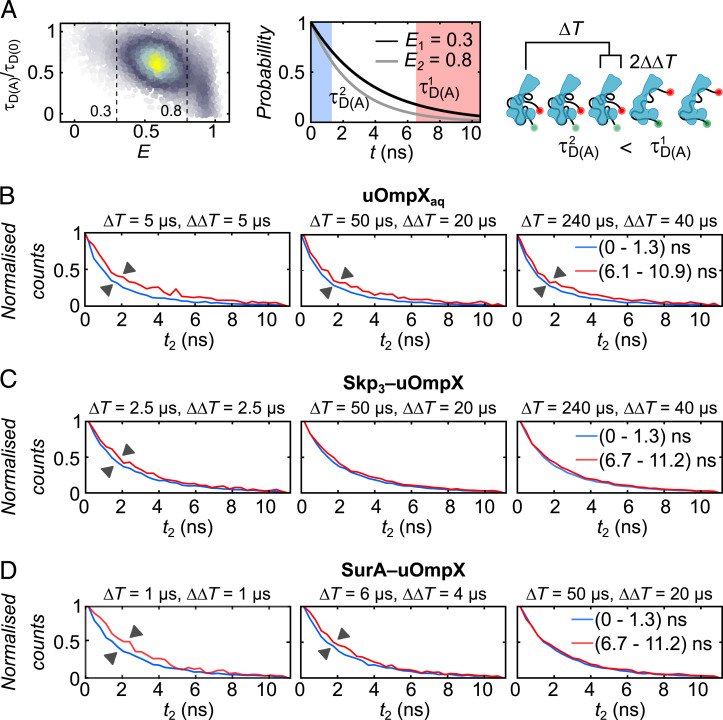
Conformational dynamics of the Skp_3_- and SurA-bound uOmpX. (*A*) Representation of species-filtered 2D FLCS using SurA–uOmpX complexes as an example. Here, *t* is the microtime of the emitted photons, Δ*T* is the time interval, and ΔΔ*T* is the window size. Emission-delay histograms corresponding to uOmpX_aq_ (*B*), Skp_3_-uOmpX (*C*), and SurA-uOmpX (*D*). The legend indicates the short and long initial microtimes.

To explore the timescale of dynamics further, we implemented species-filtered two-dimensional fluorescence lifetime correlation spectroscopy (2D FLCS) (60, 61) and extracted characteristic chain reconfiguration times. Briefly, in a first step, photon pairs of FRET efficiency-filtered fluorescence bursts with a time gap matching the time interval (Δ*T*) and a window size of 2 × ΔΔT (Fig. 3*A*) were sorted according to the microtime of the initial and final photon (*t*_1_ and *t*_2_, respectively) in the 2D emission-delay histogram (*SI Appendix*, Fig. S6). The timescale of interconversion dynamics between different molecular states were then extracted by comparing slices of the 2D histogram with either short or long initial microtimes (blue and red, respectively, in the center panel of Fig. 3*A* and in the histograms of Fig. 3 *B*–*D*). If the chosen Δ*T* matches the time of a conformational change, the emission-delay histograms for two initial microtime windows will start to separate. For uOmpX_aq_, all delay time windows Δ*T* ± ΔΔ*T* produced separated emission-delay histograms (Fig. 3*B*). The amplitude of separation was greater at a lower time point Δ*T* = 5 µs as compared to that at higher time points of 5 µs or 240 µs. This reinforced our finding that uOmpX_aq_ exhibits both fast and slow conformational dynamics by virtue of its rough energy landscape. For both Skp_3_–uOmpX (Fig. 3*C*) and SurA–uOmpX (Fig. 3*D*) complexes, by contrast, the emission-delay histograms started separating at lower time points Δ*T* = 2.5 µs and Δ*T* = 6 µs, respectively. Skp- or SurA-bound uOmpX thus shows a dominating microsecond conformational change, suggesting that both chaperones modulate the energy landscape of uOmpX such that the energy barriers between the subpopulation of their bound substrate are reduced, leading to a dynamization of the polypeptide chain in the chaperone-bound state.

To conclude, we found that binding of the chaperones Skp_3_ and SurA leads to an increased end-to-end distance of the uOmpX polypeptide chain. Skp_3_-complexed uOmpX showed a decreased conformational heterogeneity, while binding of SurA increased the conformational heterogeneity of uOmpX, indicating very distinct interaction mechanisms of the chaperones on their substrates. Complexed uOmpX exhibited fast chain reconfiguration dynamics on timescales <10 µs, unlike uOmpX_aq_ alone, which showed multitier dynamics on a large range of timescales (submillisecond to ≥100 ms) originating from its rugged energy landscape. The fast dynamics and the possible expansion of chaperone-bound uOmpX may aid in preventing uOmpX from getting trapped in a fortuitous conformation which might be folding incompetent or inaccessible to the BAM complex. A wealth of different conformations also indicates a small entropic penalty for complexing uOmpX to Skp_3_ or SurA.

### Entropic and Enthalpic Contributions of Chaperone–uOmpX Interactions.

We next set out to obtain insights into the energetic contributions of Skp and SurA chaperone–uOmpX interactions. Interaction isotherms at different temperatures allow for the extraction of enthalpic, Δ*H*, and entropic, Δ*S*, contributions to the free energy change of interaction, Δ*G*, which are connected to the association constant, *K*_a_, for a bimolecular association reaction via:

[1] $$K_{a}(T)\;=\text{exp}\left(-\frac{\Delta H}{R}\cdot \frac{1}{T}+\frac{\Delta S}{R}\right)$$

**Fig. 4.**
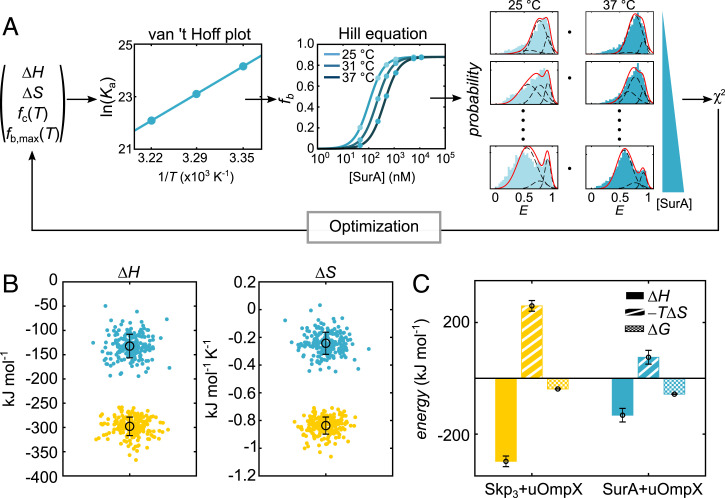
Thermodynamics of Skp_3_ and SurA interaction with uOmpX. (*A*) Schematic of the global χ^2^ minimization routine to obtain the enthalpic change (Δ*H*) and entropic change (Δ*S*) for Skp_3_ and SurA interaction with uOmpX. Δ*H*, Δ*S*, *f*_c_(*T*) (i.e., fraction of compact uOmpX at each temperature), and *f*_b,max_(*T*) (i.e., maximum fraction of Skp_3_–uOmpX or SurA–uOmpX) were the varying fitting parameters. ln(*K*_a_) is the natural logarithm of the association constant, *T* is the temperature in Kelvin (K), and [SurA] is the initial SurA concentration in nM. Protein states were modeled with three Gaussian distributions (black dashed lines) and their sum (red) compared to the experimental data. (*B*) Δ*H* and Δ*S* values for the Skp_3_ and SurA interaction with uOmpX as obtained by a bootstrapping algorithm. The best estimators for Δ*H* and Δ*S* are indicated as a black circle with SD. (*C*) Comparison of the best estimators for Δ*H, T*Δ*S*, and Δ*G* for the Skp_3_ and SurA interaction with uOmpX. Δ*G* is the free energy change of interaction at 37 °C.

In order to extract entropic and enthalpic contributions, we developed a global analysis approach that allows the reconstruction of FRET efficiency distributions at different temperatures and chaperone concentrations (Fig. 4*A* and *Methods*). Briefly, by varying the optimization parameters Δ*H* and Δ*S* and considering the fraction of the compact state (*f*_c_(*T*)), which is likely unable to be chaperone complexed, as well as the maximum fraction of the bound state (*f*_b,max_(*T*)) and minimizing the reduced χ^2^, the best estimators for Δ*H* and Δ*S* were obtained. Here, we chose a Hill coefficient of 1 for Skp_3_ and 1.5 for SurA, based on earlier reports (32, 33, 59, 62). We further assumed that Skp_3_ remained trimeric under our experimental conditions (*SI Appendix*, Methods). As *c*_1/2_ (inverse of *K*_a_) is dependent on temperature, at 37 °C, we obtained a *c*_1/2_-value of 359 ± 0.1 nM and 407 ± 0.1 nM for Skp_3_–uOmpX and SurA–uOmpX, respectively. This would convert to dissociation constants, *K*_D_ ∼ 359 nM and *K*_D_ ∼ 8.2 µM for Skp_3_–uOmpX and SurA–uOmpX, respectively. At 25 °C, we obtained a *c*_1/2_-value of 3.554 ± 0.001 nM and 102.89 ± 0.02 nM for Skp_3_–uOmpX and SurA–uOmpX, respectively. This would convert to *K*_D_ ∼ 3.6 nM and *K*_D_ ∼ 1.0 µM for Skp_3_–uOmpX and SurA–uOmpX, respectively, which is in very good agreement with previously reported values for the chaperone–OMP interactions (33, 36, 62). More interestingly, for Skp_3_–uOmpX complex formation, the binding enthalpy and entropy changes of the interaction were Δ*H* (Skp_3_–uOmpX) = –298 ± 19 kJ mol^−1^ and Δ*S* (Skp_3_–uOmpX) = –0.84 ± 0.06 kJ mol^−1^ K^−1^ (Fig. 4*B*), respectively, yielding a Δ*G* (Skp_3_–uOmpX) = (–38.3 ± 0.2) kJ mol^−1^ at 37 °C. The change of enthalpy upon binding is large, which is in agreement with recent findings demonstrating a rich interaction of the uOmpX polypeptide chain with the cavity formed by Skp_3_ (32). At the same time, the change in interaction entropy is strongly negative, suggesting a stark reduction of the overall configurational space for uOmpX upon its encapsulation by Skp_3_. Hence, Skp_3_ binding to uOmpX is entropically unfavorable and yet this entropic penalty is counterweighted by a large enthalpic contribution, enabling the binding of Skp_3_ to the client OMP.

For SurA–uOmpX complex formation, the binding enthalpy and entropy changes of interaction were Δ*H* (SurA–uOmpX) = –132 ± 24 kJ mol^−1^ and Δ*S* (SurA–uOmpX) = –0.24 ± 0.08 kJ mol^−1^ K^−1^ (Fig. 4*B*), respectively, yielding an overall stabilization of the complex of Δ*G* (SurA–uOmpX) = (–57 ± 1) kJ mol^−1^ at 37 °C, which is slightly more favorable than that for Skp_3_. The change of enthalpy was twofold lower than that for Skp_3_, likely due to more transient binding as suggested in recent studies (31, 33). At the same time Δ*S* upon SurA binding was threefold lower than with Skp_3_ and close to zero, indicating only a minimal change of configurational space for the binding of SurA to uOmpX. Although our observed interaction enthalpy and entropy are composed of multiple components (*SI Appendix*, Methods), they agree well with our finding of an increased coefficient of variation (i.e., structural heterogeneity) for SurA–uOmpX as compared to Skp_3_–uOmpX. Thus, binding of SurA to its client uOmpX is markedly different in nature than Skp_3_ binding to uOmpX. The reduction of conformational freedom of uOmpX in the cavity of Skp_3_ needs to be compensated by an increased interaction enthalpy (Fig. 4*C*). By contrast, uOmpX binding to the surface of SurA reduces the conformational freedom to a lesser extent and thus a lower enthalpic contribution is sufficient to enable binding (Fig. 4*C*).

### SurA and Skp_3_ Act Synergistically as Disaggregases on Oligomeric OmpX Structures.

Aside from the known uOMP holdase activities of Skp and SurA, recent work has suggested that Skp also has the ability to act as a disaggregase to disassemble oligomeric OMP structures (38). By contrast, only indirect evidence (18, 39) suggests that SurA might be involved in synergetic interaction with other periplasmic chaperones, such as Skp, under cellular stress in the disassembly of aggregates.

**Fig. 5.**
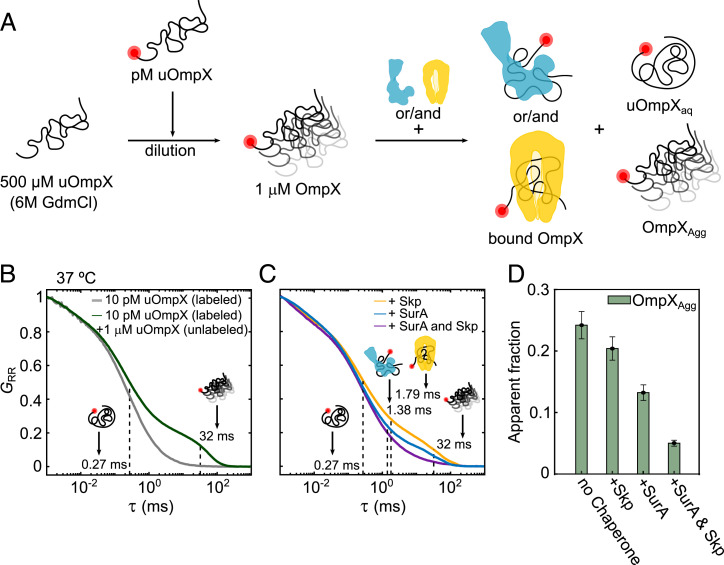
Disaggregation of OmpX aggregates by Skp and SurA. (*A*) Dilution and complex formation scheme to investigate OmpX disaggregation by Skp and SurA. (*B*) FCS curves corresponding to measurements containing 10 pM uOmpX (gray) and 1 µM uOmpX + 10 pM uOmpX (green) in aqueous buffer conditions without chaperones yield two species, namely, uOmpX_aq_ and/or OmpX_Agg_, with diffusion times as indicated. Here, *G*_RR_ is the normalized autocorrelation function of the acceptor dye and τ is the diffusion time. (*C*) Upon addition of Skp (yellow) or SurA (blue) or both (purple) to the aggregate mixture, more species appear corresponding to the Skp_3_–uOmpX and/or SurA–uOmpX complex with diffusion times as indicated. (*D*) The probability of OmpX_Agg_ (green bar) in different measurement conditions, as folllows: at a micromolar concentration of OmpX in aqueous buffer without chaperones, at a micromolar concentration of OmpX in presence of Skp (+Skp), at a micromolar concentration of OmpX in presence of SurA (+SurA), and at a micromolar concentration of OmpX in presence of both the chaperones (+ SurA and Skp).

To address this question, we investigated the action of Skp_3_ and SurA toward aggregated OmpX (OmpX_Agg_) and devised an FCS-based assay that allows us to probe the disaggregase efficacy of the two chaperones. To this extent, we diluted labeled and denatured uOmpX at pM concentrations with unlabeled and denatured uOmpX in buffer to yield a final OmpX concentration of 1 µM (Fig. 5*A*). The residual GdmCl and LDAO concentrations were 12 mM and <200 nM, respectively. It is important to note, that due to the 100,000-fold concentration difference of labeled and unlabeled uOmpX, only a small fraction of potential aggregates will be fluorescently marked and thus visible to FCS measurements. We incubated the mixture for 10 min, subsequently performed FCS measurements at 37 °C, and obtained a multimodal autocorrelation function with two major components suggesting the presence of a fast and a slowly diffusing species (Fig. 5*B*, green). A comparison with an FCS curve recorded from a highly diluted sample of only labeled uOmpX_aq_ (∼10 pM) indicates that the fast-diffusing component in the correlation curve of the aggregation sample stems from monomeric uOmpX_aq_, with a characteristic diffusion time of τ_Diff_ = (0.27 ± 0.01) ms (Fig. 5*B*, gray; *SI Appendix*, Table S6 and Fig. S7*A*). The second, slowly diffusing species in the OmpX_Agg_ measurements showed a τ_Diff_ = (31.6 ± 1.3) ms (*SI Appendix*, Fig. S7*B*), which is about two orders of magnitude slower than that of single uOmpX_aq_ molecules. Notably, OmpX_Agg_ is possibly an ensemble of differently sized aggregates; hence, values reported here reflect average values. We determined that in our aggregation mixture, about 24 ± 2% of the labeled uOmpX molecules are part of aggregates (termed apparent fraction; Fig. 4*D*). Interestingly, at lower temperatures, the propensity of OmpX aggregation was reduced (*SI Appendix*, Fig. S7*E*).

Next, we studied the effect that the chaperones Skp and SurA have on OmpX aggregation. To this end, we first incubated the mixture containing 1 µM unlabeled uOmpX and 10 pM labeled uOmpX for 10 min and then added either 2.5 µM Skp_3_ or 5.8 µM SurA to the mixture. We recorded FCS curves at 37 °C directly after the dilution and obtained multimodal curves, indicating the presence of more than two components in solution. In addition to the fast-diffusing species with τ_Diff_ = (0.27 ± 0.01) ms reflecting free uOmpX_aq_ and the aggregated OmpX_Agg_ species with very high diffusion times (τ_Diff_ ∼ (32 ± 1) ms), we expected to also observe chaperone-complexed uOmpX species with intermediate diffusion times. By comparison with an FCS curve recorded from a highly dilute sample of labeled uOmpX_aq_ (∼10 pM), we assigned diffusion times of τ_Diff_ = (1.79 ± 0.05) ms and (1.38 ± 0.03) ms to Skp_3_- and SurA-bound monomeric uOmpX, respectively (*SI Appendix*, Fig. S7 *B* and *C*). To quantify the fractional amounts of the three components (i.e., uOmpX_aq_, chaperone-bound monomeric uOmpX, and OmpX_Agg_), we fitted the autocorrelation data of our mixture experiment with three components. Here, the respective diffusion times were kept constant to extract the amplitudes of each component as they are inversely proportional to the concentrations and fractional amounts of the species. The addition of Skp_3_ to the aggregation mixture reduced the apparent fraction of OmpX_Agg_ to 20 ± 2% and the addition of SurA even to 13 ± 1%. A reduction of apparent OmpX_Agg_ was only observed at chaperone concentrations above 1 µM (*SI Appendix*, Fig. S7*F*). Interestingly, in both cases, the disaggregation of OmpX was found to be accompanied by an increase in the free uOmpX_aq_ population (*SI Appendix*, Fig. S7*G*) and only negligibly in the chaperone–uOmpX complex. Assuming uniform mixing of the labeled and unlabeled uOmpX sample, the increase of the free uOmpX population suggests that the chaperones show a higher affinity toward OmpX_Agg_ as compared to uOmpX. This finding is significant, as it suggests that the careful adaptation of affinities is crucial during physiological stress conditions. In such a scenario, disaggregation activity might be more important than holdase activity to prevent the formation of large toxic OMP aggregates in the periplasm.

Finally, we asked the question whether there are synergistic effects on the disaggregation reaction, given that both chaperones are present in the periplasm. We performed our disaggregation assay in which both chaperones were added simultaneously to the mixture containing OmpX_Agg_ and labeled uOmpX at pM concentrations after a 10-min incubation at 37 °C. Strikingly, the fraction of OmpX_Agg_ was reduced to ∼5% (Fig. 5*D*). Taking into account our single-chaperone measurements, for an independent, additive reduction of the apparent OmpX_Agg_, we would have expected a reduction to ∼9%. The increased disaggregation by a factor of two for both chaperones together suggests therefore a cooperative action of Skp_3_ and SurA. Furthermore, the additional presence of the chaperone-bound uOmpX fraction (∼8%) in the presence of both Skp and SurA could originate from handing over of uOmpX between the chaperones or from a dynamic adaptation of the chaperone functionalities between disaggregation and chaperoning activities.

## Conclusions

Periplasmic chaperones Skp and SurA are essential players in OMP biogenesis. They act as holdases, preventing uOMP substrates from misfolding and exhibit disaggregase functionalities, aiding in the clearance of OMP aggregates (32, 38). Understanding the molecular action mechanisms of these chaperones is fundamental to unraveling the functional role that these chaperones play during OMP biogenesis. Here, we have provided an intimate view on the structural, dynamic, and thermodynamic aspects of uOmpX in complex with Skp and SurA and explored their disaggregase activities.

**Fig. 6.**
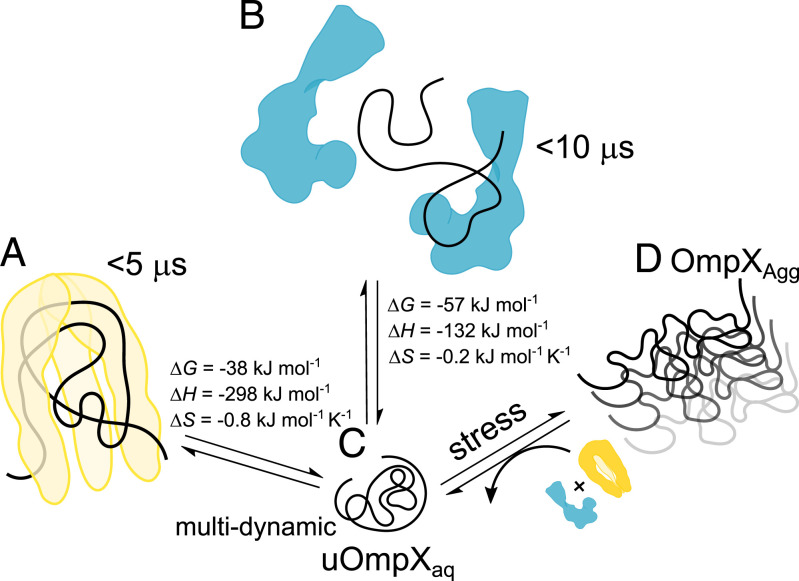
Model of Skp_3_ and SurA chaperone action on uOmpX and OmpX_Agg_. (*A*) Skp_3_–uOmpX is expanded due to numerous intermolecular interactions between the chaperone and its substrate, while the substrate itself undergoes fast chain reconfiguration on timescales <5 μs. (*B*) The increased expansion of uOmpX upon incrementing [SurA] indicates that more than one molecule of SurA binds to uOmpX. Similar to Skp_3_, SurA expands its substrate and induces uOmpX chain reconfiguration on a timescale of <10 μs. Interactions of both chaperones with uOmpX are energetically calibrated through an exquisite entropy–enthalpy compensation, as indicated by the values of entropy and enthalpy change (Δ*H* and Δ*S*, respectively), along with Δ*G*. (*C*) uOmpX_aq_ exhibits both submillisecond chain reconfiguration dynamics and ≥100 ms timescale conformational changes in aqueous buffer. (*D*) Both chaperones can disassemble aggregated OmpX, which can emerge under stress conditions.

Using smFRET, we have characterized the structural dynamics of chaperone-free and chaperone-bound uOmpX under near-native conditions and show that both chaperones Skp and SurA expand uOmpX upon binding. While previous reports have observed an expansion for SurA-bound OMPs (31, 38), an expansion upon Skp binding of an OMP in its cavity has not been observed. More surprisingly, the Skp_3_-bound uOmpX state exhibits a greater end-to-end distance as compared to the SurA–uOmpX state over the concentration range tested, suggesting that uOmpX interacts with Skp_3_ at multiple sites by spreading across the inner surface of the chaperone cavity and appears conformationally expanded within the chaperone cavity (Fig. 6*A*). The expansion of uOmpX upon Skp_3_ binding was independent of the [Skp_3_], indicating that the valency of Skp_3_ likely does not change as expected for the small eight β-stranded OmpX (59). The SurA–uOmpX complex (Fig. 6*B*), by contrast, showed an increased expansion with increasing [SurA], which is in line with multiple SurAs binding to one uOmpX. The higher valency was previously already suggested with a Hill-coefficient of *n* = 1.5 for the SurA–uOmpX association (33). Interestingly, chaperone-induced expansion was also observed for the ATP-dependent DnaJ–DnaK system for cytosolic proteins as clients (63), by forming a chain of DnaK molecules on the denatured substrate. However, unlike for Skp_3_ and SurA, for DnaK, the dynamics of interaction were largely dominated by ATP hydrolysis. Mechanistically, an expansion of OMPs by either Skp or SurA likely prevents intrachain contacts and, by extension, misfolding of uOmpX in the aqueous periplasmic space. Additionally, chain expansion can aid in exposing the β-signal peptide sequence, which facilitates recognition by BAM (64, 65). Hence, our results point to a general mechanism of chain expansion, which in the case of Skp and SurA, enables OMP chains to defer misfolding even in the absence of a high-energy substrate.

We further quantified the dynamics of both Skp_3_- and SurA-bound uOmpX using 2D-FLCS. We demonstrate that the intrachain dynamics of the chaperone-complexed uOmpX chain are on a timescale of 5 to 10 μs (Fig. 6 *A* and *B*), whereby the fast chain reconfiguration dynamics are possibly facilitated through local transient interactions with the chaperones. This is in contrast to the multitier (i.e., both slow conformational and fast reconfiguration chain) dynamics observed for the aqueous uOmpX state (Fig. 6*C*). Periplasmic chaperones thus increase conformational flexibility, despite the expansion of the unfolded state. We hypothesize that such a manipulation and dynamization of the energy landscape of the OMP substrate might be beneficial for BAM interactions (64). Notably, transient binding interactions and multivalency interactions were also observed for the cytoplasmic chaperone Trigger Factor (TF), which is structurally homologous to SurA, and like the latter is also involved in ATP-independent transfer of clients to the downstream chaperone machinery for folding (66).

Given that the periplasm is deficient of ATP as an energy source, protein folding and chaperoning, involving binding to the client uOMPs, have to be driven entirely by a change in the free energy of the system. An analysis of binding isotherms at different temperatures revealed that the change of entropy upon binding to the client uOmpX substrate was low for Skp_3_ and close to zero for SurA, suggesting that the conformational freedom is affected only minimally. A low entropic change upon binding has been hypothesized to be favorable for locally transient chaperone–uOMP interactions, due to a reduced enthalpy–entropy compensation (29). Strikingly, comparing Skp_3_ and SurA, we find that the chaperone-uOmpX interactions are fine-tuned exquisitely, meaning a higher entropic interaction change with Skp_3_ is compensated by a higher interaction enthalpic term, or vice versa in the case for SurA, to ensure a favorable, yet low change of free energy upon binding (Fig. 6). Hence, the different contributions of enthalpy and entropy to both complexes suggest that the thermodynamics of the Skp_3_–uOmpX and SurA–uOmpX interaction are differently calibrated in accordance with their chaperoning mechanisms.

Lastly, we found that both chaperones have the ability to disassemble aggregated OmpX (Fig. 6*D*). Interestingly, both chaperones show a higher affinity toward aggregated OmpX than uOmpX. A higher affinity toward the aggregate might be particularly useful when aggregated OmpX emerges, for example, under stress conditions to avoid the accumulation of OMP aggregates. Surprisingly, we found that Skp_3_ and SurA act synergistically on uOmpX aggregates, suggesting higher order interactions between both periplasmic chaperones. Disaggregation mechanisms have also been observed in the ATP-dependent cytoplasmic chaperones of the Hsp70 family (67, 68). However, both Skp and SurA disaggregate oligomeric structures in the absence of an external energy source, thereby supporting the known disaggregase DegP, with a mechanism that is yet to be determined. In future experiments, it will be interesting to dissect more closely the mechanism of OMP oligomerization, how these states are disassembled, and how oligomerization impacts protein conformation.

In summary, both Skp and SurA binding to uOmpX expands the unfolded polypeptide chain distinctly. Interestingly, the unfolded polypeptide chain, in complex with both chaperones, exhibits a dynamization on the microsecond timescale. While the structural features of the bound polypeptide appear similar, the thermodynamic aspects of the Skp_3_–uOmpX and SurA–uOmpX interaction differ and SurA binding results in a reduced loss of entropy compared to Skp binding. In a biological context, expansion and fast chain reconfiguration of the bound polypeptide may serve to ensure that the uOMP remains accessible to the BAM complex. The enthalpy–entropy compensations consent with the binding modes of the two chaperones to maintain an energetically favorable interaction with uOmpX. Lastly, both chaperones synergistically disaggregate OmpX aggregates, suggesting additional interactions between both chaperones that enhance the disaggregation. We hypothesize that the tightly regulated multifaceted functionalities of both chaperones enable cellular vitality to be maintained and regulated under normal and stress conditions.

## Methods

### Protein Production.

A tag-free double-cysteine variant of OmpX (OmpX_1,149_) without a signal sequence was expressed as inclusion bodies in *Escherichia coli* BL21(DE3) cells and purified following standard procedures including anion exchange chromatography, as previously reported (69, 70). After refolding, the protein was labeled with FRET donor (ATTO532, Atto-Tec) and acceptor (Abberior STAR 635P, Abberior) dyes. We further produced two other OmpX variants; however, they were not used for the main study due to their misbehavior possibly caused by higher propensity toward aggregation (*SI Appendix*, Fig. S8). Details are given in *SI Appendix*.

Skp and SurA were produced as N-terminal hexahistidine (His_6_) fusion proteins in *E. coli* BL21 (DE3) cells and purified using immobilized metal affinity chromatography under denaturing conditions, as previously reported (32, 59). The proteins were refolded prior to experiments. Details on the production and purification are given in *SI Appendix*.

### Sample Preparation for Single-Molecule Measurements.

A multistep dilution protocol, as shown in Fig. 1*A*, was followed to prepare samples for smFRET and FCS measurements. In both FCS and smFRET experiments, the labeled protein was present at a concentration of 20 pM in buffer containing 20 mM Tris⋅HCl (pH 8.0) and 150 mM NaCl. The remaining concentration of GdmCl and LDAO was 6 mM and 87.5 nM, respectively. For FCS experiments involving OmpX aggregates, as shown in Fig. 5*A*, the concentration of GdmCl and LDAO was 12 mM and 175 nM, respectively, after addition of 1 μM uOmpX in the same buffer as that used for smFRET experiments. Details are given in *SI Appendix*. Melting curves of labeled and unlabeled OmpX obtained through circular dichroism spectroscopy measurements overlapped within error indicating that labeling did not affect the protein stability markedly (*SI Appendix*, Fig. S9*A*).

### smFRET and FCS Measurements.

Experiments were carried out using a single-molecule confocal fluorescence microscope as previously described (71) and detailed in *SI Appendix*. Data analysis was performed with custom-written Matlab scripts (Mathworks). Single-molecule events were identified from the acquired photon stream by a burst search algorithm. Details about smFRET and FCS analysis procedures, including burst selection, data reduction, calculation of FRET efficiencies, and fluorescence, are given in *SI Appendix*.

## Supplementary Material

Supplementary File

## Data Availability

Raw ht3 data (time-correlated single-photon counting data) and preanalyzed data from our single-molecule FRET and FCS experiments data have been deposited in a publicly accessible database, OpARA (DOI: http://dx.doi.org/10.25532/OPARA-162).

## References

[1] F. U. Hartl, M. Hayer-Hartl, Molecular chaperones in the cytosol: From nascent chain to folded protein. Science 295, 1852–1858 (2002). 11884745 10.1126/science.1068408

[2] Y. E. Kim, M. S. Hipp, A. Bracher, M. Hayer-Hartl, F. U. Hartl, Molecular chaperone functions in protein folding and proteostasis. Annu. Rev. Biochem. 82, 323–355 (2013). 23746257 10.1146/annurev-biochem-060208-092442

[3] A. Mogk, C. Ruger-Herreros, B. Bukau, Cellular functions and mechanisms of action of small heat shock proteins. Annu. Rev. Microbiol. 73, 89–110 (2019). 31091419 10.1146/annurev-micro-020518-115515

[4] S. E. Rollauer, M. A. Sooreshjani, N. Noinaj, S. K. Buchanan, Outer membrane protein biogenesis in Gram-negative bacteria. Philos. Trans. R. Soc. Lond. B Biol. Sci. 370, 20150023 (2015). 26370935 10.1098/rstb.2015.0023 PMC4632599

[5] E. R. Green, J. Mecsas, Bacterial secretion systems: An overview. Microbiol. Spectr. 4, 10.1128/microbiolspec.VMBF-0012-2015 (2016). 10.1128/microbiolspec.VMBF-0012-2015 PMC4804464 26999395

[6] A. E. Rizzitello, J. R. Harper, T. J. Silhavy, Genetic evidence for parallel pathways of chaperone activity in the periplasm of *Escherichia coli*. J. Bacteriol. 183, 6794–6800 (2001). 11698367 10.1128/JB.183.23.6794-6800.2001 PMC95519

[7] A. M. Plummer, K. G. Fleming, From chaperones to the membrane with a BAM! Trends Biochem. Sci. 41, 872–882 (2016). 27450425 10.1016/j.tibs.2016.06.005 PMC5420074

[8] K. Kim , Outer membrane proteins A (OmpA) and X (OmpX) are essential for basolateral invasion of Cronobacter sakazakii. Appl. Environ. Microbiol. 76, 5188–5198 (2010). 20543055 10.1128/AEM.02498-09 PMC2916488

[9] G. E. Schulz, β-Barrel membrane proteins. Curr. Opin. Struct. Biol. 10, 443–447 (2000). 10981633 10.1016/s0959-440x(00)00120-2

[10] R. Koebnik, K. P. Locher, P. Van Gelder, Structure and function of bacterial outer membrane proteins: Barrels in a nutshell. Mol. Microbiol. 37, 239–253 (2000). 10931321 10.1046/j.1365-2958.2000.01983.x

[11] M. Braun, T. J. Silhavy, Imp/OstA is required for cell envelope biogenesis in *Escherichia coli*. Mol. Microbiol. 45, 1289–1302 (2002). 12207697 10.1046/j.1365-2958.2002.03091.x

[12] E. M. Hart , A small-molecule inhibitor of BamA impervious to efflux and the outer membrane permeability barrier. Proc. Natl. Acad. Sci. U.S.A. 116, 21748–21757 (2019). 31591200 10.1073/pnas.1912345116 PMC6815139

[13] Y. Imai , A new antibiotic selectively kills Gram-negative pathogens. Nature 576, 459–464 (2019). 31747680 10.1038/s41586-019-1791-1 PMC7188312

[14] A. Luther , Chimeric peptidomimetic antibiotics against Gram-negative bacteria. Nature 576, 452–458 (2019). 31645764 10.1038/s41586-019-1665-6

[15] S. A. Paschen , Evolutionary conservation of biogenesis of β-barrel membrane proteins. Nature 426, 862–866 (2003). 14685243 10.1038/nature02208

[16] L. M. McMorran, D. J. Brockwell, S. E. Radford, Mechanistic studies of the biogenesis and folding of outer membrane proteins in vitro and in vivo: What have we learned to date? Arch. Biochem. Biophys. 564, 265–280 (2014). 24613287 10.1016/j.abb.2014.02.011 PMC4262575

[17] L. K. Tamm, H. Hong, B. Liang, Folding and assembly of beta-barrel membrane proteins. Biochim. Biophys. Acta 1666, 250–263 (2004). 15519319 10.1016/j.bbamem.2004.06.011

[18] S. M. Costello, A. M. Plummer, P. J. Fleming, K. G. Fleming, Dynamic periplasmic chaperone reservoir facilitates biogenesis of outer membrane proteins. Proc. Natl. Acad. Sci. U.S.A. 113, E4794–E4800 (2016). 27482090 10.1073/pnas.1601002113 PMC4995976

[19] K. Denoncin, J. Schwalm, D. Vertommen, T. J. Silhavy, J.-F. Collet, Dissecting the *Escherichia coli* periplasmic chaperone network using differential proteomics. Proteomics 12, 1391–1401 (2012). 22589188 10.1002/pmic.201100633 PMC3883104

[20] J. C. Malinverni, T. J. Silhavy, Assembly of outer membrane β-barrel proteins: The Bam Complex. Ecosal Plus 4, 10.1128/ecosalplus.4.3.8. (2011). PMC4231818 26442509

[21] C. Wülfing, A. Plückthun, Protein folding in the periplasm of *Escherichia coli*. Mol. Microbiol. 12, 685–692 (1994). 8052121 10.1111/j.1365-2958.1994.tb01056.x

[22] T. A. Walton, C. M. Sandoval, C. A. Fowler, A. Pardi, M. C. Sousa, The cavity-chaperone Skp protects its substrate from aggregation but allows independent folding of substrate domains. Proc. Natl. Acad. Sci. U.S.A. 106, 1772–1777 (2009). 19181847 10.1073/pnas.0809275106 PMC2644113

[23] E. Bitto, D. B. McKay, Crystallographic structure of SurA, a molecular chaperone that facilitates folding of outer membrane porins. Structure 10, 1489–1498 (2002). 12429090 10.1016/s0969-2126(02)00877-8

[24] S. W. Lazar, R. Kolter, SurA assists the folding of *Escherichia coli* outer membrane proteins. J. Bacteriol. 178, 1770–1773 (1996). 8626309 10.1128/jb.178.6.1770-1773.1996 PMC177866

[25] R. Chen, U. Henning, A periplasmic protein (Skp) of *Escherichia coli* selectively binds a class of outer membrane proteins. Mol. Microbiol. 19, 1287–1294 (1996). 8730870 10.1111/j.1365-2958.1996.tb02473.x

[26] G. Mas, S. Hiller, Conformational plasticity of molecular chaperones involved in periplasmic and outer membrane protein folding. FEMS Microbiol. Lett. 365, fny121 (2018). 10.1093/femsle/fny121 29893830

[27] K. G. Fleming, A combined kinetic push and thermodynamic pull as driving forces for outer membrane protein sorting and folding in bacteria. Philos. Trans. R. Soc. Lond. B Biol. Sci. 370, 20150026 (2015). 26370938 10.1098/rstb.2015.0026 PMC4632602

[28] S. Wu , Interaction between bacterial outer membrane proteins and periplasmic quality control factors: A kinetic partitioning mechanism. Biochem. J. 438, 505–511 (2011). 21671888 10.1042/BJ20110264

[29] S. Hiller, Chaperone-bound clients: The importance of being dynamic. Trends Biochem. Sci. 44, 517–527 (2019). 30611607 10.1016/j.tibs.2018.12.005

[30] J. G. Sklar, T. Wu, D. Kahne, T. J. Silhavy, Defining the roles of the periplasmic chaperones SurA, Skp, and DegP in *Escherichia coli*. Genes Dev. 21, 2473–2484 (2007). 17908933 10.1101/gad.1581007 PMC1993877

[31] D. C. Marx , SurA is a cryptically grooved chaperone that expands unfolded outer membrane proteins. Proc. Natl. Acad. Sci. U.S.A. 117, 28026–28035 (2020). 33093201 10.1073/pnas.2008175117 PMC7668074

[32] B. M. Burmann, C. Wang, S. Hiller, Conformation and dynamics of the periplasmic membrane-protein-chaperone complexes OmpX-Skp and tOmpA-Skp. Nat. Struct. Mol. Biol. 20, 1265–1272 (2013). 24077225 10.1038/nsmb.2677

[33] A. N. Calabrese , Inter-domain dynamics in the chaperone SurA and multi-site binding to its outer membrane protein clients. Nat. Commun. 11, 2155 (2020). 32358557 10.1038/s41467-020-15702-1 PMC7195389

[34] G. Krainer , Slow interconversion in a heterogeneous unfolded-state ensemble of outer-membrane phospholipase A. Biophys. J. 113, 1280–1289 (2017). 28629619 10.1016/j.bpj.2017.05.037 PMC5607043

[35] C. P. Moon, N. R. Zaccai, P. J. Fleming, D. Gessmann, K. G. Fleming, Membrane protein thermodynamic stability may serve as the energy sink for sorting in the periplasm. Proc. Natl. Acad. Sci. U.S.A. 110, 4285–4290 (2013). 23440211 10.1073/pnas.1212527110 PMC3600475

[36] J. Qu, C. Mayer, S. Behrens, O. Holst, J. H. Kleinschmidt, The trimeric periplasmic chaperone Skp of Escherichia coli forms 1:1 complexes with outer membrane proteins via hydrophobic and electrostatic interactions. J. Mol. Biol. 374, 91–105 (2007). 17928002 10.1016/j.jmb.2007.09.020

[37] B. Schiffrin , Effects of periplasmic chaperones and membrane thickness on BamA-catalyzed outer-membrane protein folding. J. Mol. Biol. 429, 3776–3792 (2017). 28919234 10.1016/j.jmb.2017.09.008 PMC5692476

[38] G. Li , Single-molecule detection reveals different roles of Skp and SurA as chaperones. ACS Chem. Biol. 13, 1082–1089 (2018). 29543429 10.1021/acschembio.8b00097

[39] A. P. Chum, S. R. Shoemaker, P. J. Fleming, K. G. Fleming, Plasticity and transient binding are key ingredients of the periplasmic chaperone network. Protein Sci., pro.3641 (2019). 10.1002/pro.3641 PMC6566527 31074917

[40] B. Schiffrin, D. J. Brockwell, S. E. Radford, Outer membrane protein folding from an energy landscape perspective. BMC Biol. 15, 123 (2017). 29268734 10.1186/s12915-017-0464-5 PMC5740924

[41] J. E. Horne, S. E. Radford, A growing toolbox of techniques for studying β-barrel outer membrane protein folding and biogenesis. Biochem. Soc. Trans. 44, 802–809 (2016). 27284045 10.1042/BST20160020 PMC4900752

[42] R. E. Jefferson, D. Min, K. Corin, J. Y. Wang, J. U. Bowie, Applications of single-molecule methods to membrane protein folding studies. J. Mol. Biol. 430, 424–437 (2018). 28549924 10.1016/j.jmb.2017.05.021 PMC5700846

[43] G. Krainer, S. Keller, M. Schlierf, Structural dynamics of membrane-protein folding from single-molecule FRET. Curr. Opin. Struct. Biol. 58, 124–137 (2019). 31323499 10.1016/j.sbi.2019.05.025

[44] G. Krainer , Ultrafast protein folding in membrane-mimetic environments. J. Mol. Biol. 430, 554–564 (2018). 29128595 10.1016/j.jmb.2017.10.031

[45] H. Hofmann , Single-molecule spectroscopy of protein folding in a chaperonin cage. Proc. Natl. Acad. Sci. U.S.A. 107, 11793–11798 (2010). 20547872 10.1073/pnas.1002356107 PMC2900638

[46] G. Krainer , CFTR transmembrane segments are impaired in their conformational adaptability by a pathogenic loop mutation and dynamically stabilized by Lumacaftor. J. Biol. Chem. 295, 1985–1991 (2020). 31882543 10.1074/jbc.AC119.011360 PMC7029128

[47] B. Schuler, H. Hofmann, Single-molecule spectroscopy of protein folding dynamics—Expanding scope and timescales. Curr. Opin. Struct. Biol. 23, 36–47 (2013). 23312353 10.1016/j.sbi.2012.10.008

[48] B. Hellenkamp , Precision and accuracy of single-molecule FRET measurements—A multi-laboratory benchmark study. Nat. Methods 15, 669–676 (2018). 30171252 10.1038/s41592-018-0085-0 PMC6121742

[49] A. Hoffmann , Quantifying heterogeneity and conformational dynamics from single molecule FRET of diffusing molecules: Recurrence analysis of single particles (RASP). Phys. Chem. Chem. Phys. 13, 1857–1871 (2011). 21218223 10.1039/c0cp01911a PMC3378030

[50] H. Tafer, S. Hiller, C. Hilty, C. Fernández, K. Wüthrich, Nonrandom structure in the urea-unfolded Escherichia coli outer membrane protein X (OmpX). Biochemistry 43, 860–869 (2004). 14744128 10.1021/bi0356606

[51] B. Schuler, A. Soranno, H. Hofmann, D. Nettels, Single-molecule FRET spectroscopy and the polymer physics of unfolded and intrinsically disordered proteins. Annu. Rev. Biophys. 45, 207–231 (2016). 27145874 10.1146/annurev-biophys-062215-010915

[52] K. Henzler-Wildman, D. Kern, Dynamic personalities of proteins. Nature 450, 964–972 (2007). 18075575 10.1038/nature06522

[53] E. Frotscher, G. Krainer, A. Hartmann, M. Schlierf, S. Keller, Conformational dynamics govern the free-energy landscape of a membrane-interacting protein. ACS Omega 3, 12026–12032 (2018). 31459283 10.1021/acsomega.8b01609 PMC6690567

[54] E. S. O’Brien , Membrane proteins have distinct fast internal motion and residual conformational entropy. Angew. Chem. Int. Ed. Engl. 59, 11108–11114 (2020). 32277554 10.1002/anie.202003527 PMC7318686

[55] G. Mas , Regulation of chaperone function by coupled folding and oligomerization. Sci. Adv. 6, eabc5822 (2020). 33087350 10.1126/sciadv.abc5822 PMC7577714

[56] D. A. Holdbrook , A spring-loaded mechanism governs the clamp-like dynamics of the Skp chaperone. Structure 25, 1079–1088 (2017). 28648612 10.1016/j.str.2017.05.018

[57] C. W. Sandlin, N. R. Zaccai, K. G. Fleming, Skp trimer formation is insensitive to salts in the physiological range. Biochemistry 54, 7059–7062 (2015). 26579730 10.1021/acs.biochem.5b00806 PMC4905700

[58] T. Masuda, N. Saito, M. Tomita, Y. Ishihama, Unbiased quantitation of *Escherichia coli* membrane proteome using phase transfer surfactants. Mol. Cell. Proteomics 8, 2770–2777 (2009). 19767571 10.1074/mcp.M900240-MCP200 PMC2816013

[59] B. Schiffrin , Skp is a multivalent chaperone of outer-membrane proteins. Nat. Struct. Mol. Biol. 23, 786–793 (2016). 27455461 10.1038/nsmb.3266 PMC5018216

[60] K. Ishii, T. Tahara, Two-dimensional fluorescence lifetime correlation spectroscopy. 2. Application. J. Phys. Chem. B 117, 11423–11432 (2013). 23977902 10.1021/jp406864e

[61] K. Ishii, T. Tahara, Two-dimensional fluorescence lifetime correlation spectroscopy. 1. Principle. J. Phys. Chem. B 117, 11414–11422 (2013). 23977832 10.1021/jp406861u

[62] J. R. Humes , The role of SurA PPIase domains in preventing aggregation of the outer-membrane proteins tOmpA and OmpT. J. Mol. Biol. 431, 1267–1283 (2019). 30716334 10.1016/j.jmb.2019.01.032 PMC7618261

[63] R. Kellner , Single-molecule spectroscopy reveals chaperone-mediated expansion of substrate protein. Proc. Natl. Acad. Sci. U.S.A. 111, 13355–13360 (2014). 25165400 10.1073/pnas.1407086111 PMC4169939

[64] M. W. Franklin , Evolutionary pathways of repeat protein topology in bacterial outer membrane proteins. eLife 7, e40308 (2018). 30489257 10.7554/eLife.40308 PMC6340704

[65] V. Robert , Assembly factor Omp85 recognizes its outer membrane protein substrates by a species-specific C-terminal motif. PLoS Biol. 4, e377 (2006). 17090219 10.1371/journal.pbio.0040377 PMC1634882

[66] A. Hoffmann, B. Bukau, G. Kramer, Structure and function of the molecular chaperone Trigger Factor. Biochim Biophys Acta 1803, 650–661 (2010). 20132842 10.1016/j.bbamcr.2010.01.017

[67] M. M. Schneider The Hsc70 disaggregation machinery removes monomer units directly from α-Synuclein fibril ends. *Nat. Comm. ***12**, 5999 (2021). 10.1038/s41467-021-25966-w PMC8516981 34650037

[68] A. Mogk, B. Bukau, H. H. Kampinga, Cellular handling of protein aggregates by disaggregation machines. Mol. Cell 69, 214–226 (2018). 29351843 10.1016/j.molcel.2018.01.004

[69] E. Frotscher , A fluorinated detergent for membrane-protein applications. Angew. Chem. Int. Ed. Engl. 54, 5069–5073 (2015). 25753129 10.1002/anie.201412359

[70] M. Herrmann, B. Danielczak, M. Textor, J. Klement, S. Keller, Modulating bilayer mechanical properties to promote the coupled folding and insertion of an integral membrane protein. Eur. Biophys. J. 44, 503–512 (2015). 26016666 10.1007/s00249-015-1032-y

[71] A. Hartmann, G. Krainer, S. Keller, M. Schlierf, Quantification of millisecond protein-folding dynamics in membrane-mimetic environments by single-molecule Förster resonance energy transfer spectroscopy. Anal. Chem. 87, 11224–11232 (2015). 26457727 10.1021/acs.analchem.5b03207

